# Assessment of sleep in patients with fibromyalgia: qualitative development of the fibromyalgia sleep diary

**DOI:** 10.1186/s12955-014-0111-6

**Published:** 2014-07-14

**Authors:** Leah Kleinman, Sally Mannix, Lesley M Arnold, Claire Burbridge, Kellee Howard, Kelly McQuarrie, Verne Pitman, Malca Resnick, Tom Roth, Tara Symonds

**Affiliations:** 1 Evidera, Bethesda, Maryland, USA; 2 University of Cincinnati College of Medicine, Cincinnati, Ohio, USA; 3 Pfizer Ltd, Walton Oaks, Dorking Road, Walton on the Hill, Surrey KT20 7NS, Tadworth, UK; 4 With United BioSource Corporation at the time of the study, Topeka, USA; 5 Pfizer Inc, New York, New York, USA; 6 Pfizer Inc, Groton, Connecticut, USA; 7 Henry Ford Health System, Detroit, Michigan, USA

**Keywords:** Fibromyalgia, Sleep, Qualitative, Patient, Diary

## Abstract

**Objectives:**

Sleep disturbance is a common experience in fibromyalgia (FM). The field lacks a sleep specific patient reported outcome (PRO) measure developed and validated in a FM population. The study objective is to gain an in-depth understanding of sleep in FM and to develop a PRO measure of it.

**Methods:**

Research involved the following stages: 1) A literature review conducted to identify key concepts associated with FM patient experience of sleep and PRO measures that have been used to assess this; 2) Qualitative interviews with therapeutic area experts; 3) Focus groups with FM patients who experienced sleep disturbance; 4) Development of a conceptual framework and the Fibromyalgia Sleep Diary (FMSD); and 5) Cognitive interviews with patients to explore content validity of the FMSD.

**Results:**

The literature review and expert interviews supported sleep disturbance being an important aspect of the FM patient experience, and underscored the need for a new FM specific sleep PRO measure. Results from the focus groups demonstrated that FM patients experience sleep disturbances that they attribute to their FM symptoms, such as pain and stiffness, confirming the importance of understanding more about sleep changes. Aspects of sleep raised by FM patients included poor sleep quality and insufficient quantity including difficulty with falling asleep, getting comfortable, and staying asleep; restlessness; light sleep; not feeling rested upon awakening; and difficulty starting the day. Cognitive interview results showed that the 8-item FMSD, developed to reflect the concepts identified above, was relevant to FM patients with content that was interpreted as intended.

**Conclusions:**

The FMSD was developed in line with the recommendations of the FDA PRO guidance and ISPOR PRO Task Force. The qualitative evidence generated thus far strongly supports the content validity of the FMSD as a PRO measure of sleep disturbance in FM populations. Psychometric evaluation of the FMSD to demonstrate reliability, validity and sensitivity to change is recommended as a next step.

## Introduction and background

Fibromyalgia (FM) is characterized by chronic widespread pain and tenderness [[1]]. Common associated symptoms include fatigue, mood disturbance, and sleep problems [[2]]. In a patient Delphi Panel run as part of the Outcome Measures in Rheumatology (OMERACT) project, sleep problems appeared as the fourth most important domain to FM patients with 92% of patients reporting that this domain should be assessed in FM clinical trials [[2],[3]]. In another study, published by Arnold and colleagues [[4]], patients with FM reported that disrupted sleep was a common symptom associated with FM. Most patients indicated that both fatigue and pain were directly related to the poor quality of their sleep. In OMERACT 9 (the 9th annual meeting) [[2]], sleep disturbance was noted as one of a core set of domains considered essential for assessment in FM clinical trials. The impact FM has on sleep was defined in OMERACT as difficulty falling asleep, staying asleep and unrefreshing sleep [[2]].

The Food and Drug Administration (FDA) issued a guidance document describing the necessary evidence for a Patient Reported Outcome (PRO) instrument to support label claims [[5]], which includes the evaluation of content validity as a key recommendation (i.e. assessment and documentation of how the PRO measures the concepts relevant to the population). Recently, the International Society for Pharmacoeconomics and Outcomes Research (ISPOR) PRO Task Force published five “good research practices” that are crucial steps in establishing and documenting the evidence of content validity for new PRO instruments [[6]]. These are: 1) identification of the PRO measurement concept and the context of use; 2) development of the qualitative concept elicitation protocol; 3) qualitative concept elicitation data collection among the population of interest (i.e. a pre-specified patient population); 4) analysis of the qualitative data; and 5) documentation of methods used and results of the validity assessment of the content of the PRO measure (e.g., instructions, items, response options, recall period). Current standards focus on the need for concrete evidence of the direct link between the patient perspective and the concept and item coverage in a PRO measure.

**Figure 1:**
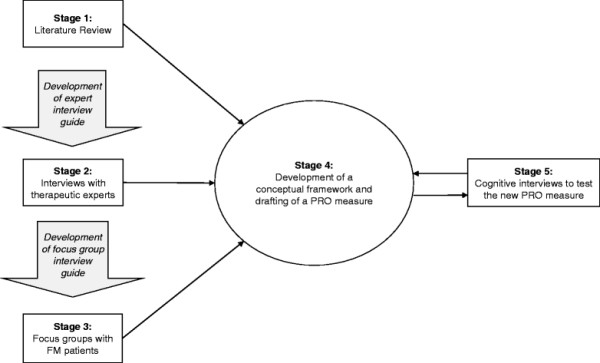
Study flow diagram.

The objective of this research study was to gain an in-depth understanding of sleep in FM and to develop a PRO measure of sleep disturbance in FM. These objectives were met by employing a multi-staged approach that encompassed qualitative and instrument development work that is in line with current PRO instrument development recommendations [[6],[7]]. This included the following stages: 1) a literature review; 2) interviews with therapeutic experts; 3) focus groups with FM patients; 4) development of a conceptual framework and drafting of a PRO measure; 5) cognitive interviews to test the new PRO measure. Each stage provided data that informed subsequent stages (see Figure 1). Each stage is presented in turn with the rationale, methods, and results.

### Stage 1 Literature review

#### Rationale

A targeted literature review was conducted to identify types of sleep disturbances in FM and any existing PRO measures used to evaluate this concept in the FM population. Evidence from the literature review was used to determine the need for a new measure of sleep in FM, to develop qualitative interview guides for the interviews with experts and the focus groups with FM patients, as well as to inform the conceptual framework.

#### Methods

The literature review was conducted in MEDLINE and EMBASE. Terms used in the search included terms for fibromyalgia and sleep disorders combined with a standard combination used to identify PRO measures (such as interview, questionnaire, rating scale, instrument, outcomes). In addition, these searches were supplemented by a review of FM or sleep-specific PRO measures listed in the PRO research database PROQOLID, as well as a review of clinical trials that targeted adult FM patients, assessed sleep outcomes, and were listed in the clinical trial search engine provided by the U.S National Institutes of Health (NIH; http://clinicaltrials.gov).

The search terms can be found in the Additional file 1 online.

#### Results

Four hundred and forty-two abstracts were reviewed for relevance. Excluded abstracts consisted of those that did not discuss key concepts related to sleep and FM, or included pediatric patients as all or part of the population. Seventy-nine articles were retrieved for full text review and categorized as observational studies (n = 25), review articles (n = 21), case–control studies (n = 10), instrument evaluations (n = 9), qualitative studies (n = 6), cross-sectional studies (n = 3), clinical trials (n = 4), and a pooled analysis (n = 1). Additionally, a search of the website clinicaltrials.gov identified 45 ongoing clinical trials in FM that included a sleep outcome used in a review of available PRO measures.

The text of the 79 articles was reviewed to identify mentions of constructs for sleep and other fibromyalgia symptoms. Across the articles reviewed, the sleep specific concepts most frequently noted as being a component of FM (i.e., mentioned in >20% of the 79 articles) included: sleep disturbance (characterized as arousals, sleep stage changes, wake after sleep onset) [[8]]; poor quality of sleep [[9],[10]]; insufficient sleep duration or quality [[11]]; daytime dysfunction [[12]]; awakening unrefreshed [[13]]; and low sleep efficiency [[14]]. Other sleep specific concepts that were mentioned less frequently included difficulty getting comfortable, habitual snoring, and daytime somnolence. The six qualitative studies reviewed endorsed sleep disturbance being part of the FM patient experience [[3],[4],[15]–[18]].

In addition, a wide variety of PRO measures that have been used to assess sleep in FM populations were identified. The most frequently utilized were the sleep item of the Fibromyalgia Impact Questionnaire (FIQ), a FM-specific measure [[19]]; the Pittsburgh Sleep Quality Index (PSQI) [[20]]; the MOS-Sleep Scale [[21]]; the Epworth Sleepiness Scale [[22]]; and the Jenkins Scale for Sleep [[23]]. Results for the sleep outcomes included in the 45 clinical trials listed on the NIH search engine were similar to the results of the literature review; 26 trials included the FIQ, 11 trials included the MOS sleep scale, seven trials included the Multidimensional Fatigue Inventory [[24]], and four trials included the PSQI. In addition the use of a daily sleep log diary was often reported, particularly in the clinical trials.

The history of the sleep specific PRO measures was explored to ascertain if any met the FDA guidance requirements for PRO development [[5]], including qualitative research conducted in the target population (i.e., patients with both FM and sleep disturbances included in both concept elicitation and cognitive interviewing), evidence of final content validity determined through quantitative analyses (e.g., Rasch models, confirmatory factor analysis), adequate psychometric characteristics, and score sensitivity to change and interpretability. The search revealed that none of the sleep specific or more generic PRO measures mentioned above had development and validation information available to demonstrate their relevance as a sleep measurement tool specifically for the FM population. The one general FM measure (FIQ) was excluded as it does not focus specifically on sleep. Thus, based on this literature review and the current standards in the field for development of PRO measures, it became apparent that, despite the importance of sleep disturbance in FM, the field was lacking a sleep specific measure that has been developed and validated for FM populations.

### Stage 2 - Interviews with therapeutic area experts

#### Rationale

Clinical input about the condition or disease area is recommended [[5]] to gather information about the clinical aspects of the condition as well as providing a starting point for discussions with patients. Interviews with four therapeutic area experts in FM were conducted to confirm the key findings of the literature review, and to provide information around how patients with FM discuss their sleep issues. This information was then used to inform the interview guide for the focus groups with FM patients. In addition to completing formal interviews with experts, two co-authors (LMA and TR) were involved in study decisions on an on-going basis.

#### Methods

A pool of US-based experts was sent a recruitment e-mail with an overview of the study and information on the role of therapeutic area experts. The e-mail requested that interested parties return a background questionnaire that asked about degree, primary clinical specialty, number of years in practice, percentage of time spent in clinical practice, and number of FM patients seen per week. The experts were then selected to participate in the one-time interview based on level of experience with treating the population of interest, as determined by responses to the background questionnaire as well as their interest and availability. A semi-structured interview guide was used to facilitate the expert interviews, which focused on topics such as impact of fibromyalgia on sleep, and impact of sleep disturbance on FM patient’s daytime function. They were also asked about experiences with assessing sleep using quantifiable instruments. Interviews were audio-recorded, and summary tables were created by listening to the audio-recorded interviews and summarizing the responses for each expert. These tables were manually reviewed to analyze the feedback across interviews and identify key themes.

#### Results

Four experts completed the one-on-one telephone interviews. They comprised three physicians (a psychiatrist, a rheumatologist and a neurologist with a specialty in sleep medicine) and one registered nurse. They had an average of 14.5 years of experience working with patients who have FM (range: 3 to 25 years), reported seeing approximately 25–30 FM patients per week (range: 10–60), and reported practicing in multiple settings, including private and academic clinical practices and research clinics (e.g., a sleep laboratory).

Experts reported that a substantial number of FM patients spontaneously raise the issue of disturbed sleep. When not specifically raised by the patients, all clinicians reported probing to see if sleep disturbance is a problem. Not surprisingly the experts used more technical descriptions (e.g., non-restorative sleep, delayed sleep onset, sleep disturbance or inability to sleep, feeling un-refreshed), but their patients use a variety of descriptive expressions such as, feeling exhausted, not having a good night’s sleep or a deep/restful sleep, awakening during the night due to light sleep, and having difficulty falling back asleep once awake. They noted that patients often differentiated between sleep quality and sleep quantity with the former being more descriptive (i.e., disturbed sleep, poor quality sleep, a feeling of being non-rested) and the latter being more quantitative (i.e., the number of hours a patient reported sleeping or being in bed, number of awakenings).

The expert panel reported using PRO measures of pain and sleep in order to quantify patient issues, or as part of the requirements for clinical trials. For example, a 0 to 10 visual analogue scale on sleep quality, the Epworth Sleepiness Questionnaire, and the SF-36 (although not a sleep specific measure) were identified; however, experts noted that these were not specific to FM, nor were they consistently effective in clinical trials or practical in clinical practice either because of lack of specificity or being cumbersome to use and score.

In summary, the findings from this portion of the research supported sleep disturbance being an important aspect of FM that would benefit from measurement tools that are more focused on the FM patients’ experience of sleep.

### Stage 3 - Focus groups

#### Rationale

Development of a PRO measure must be based on patients’ perceptions or thoughts around the concept of interest; this is the direct link from the patient voice to the measure [[5]]. Focus groups provide an opportunity to gather qualitative data in a conversational setting, allowing for group dynamics to facilitate a broad-ranging discussion in the population of interest.

#### Methods

Five focus groups consisting of a total of 36 patients with FM and documented sleep disturbance associated with FM (as noted via chart review and verification by the clinical site during recruitment), were held at three different community-based clinical sites in the United States including two in California and one in Texas. The sample was a convenience sample with sites recruiting participants using eligibility criteria designed to align with key criteria in the study sponsor’s FM clinical trial populations. This study was reviewed and approved by an IRB (Ethical and Independent Review Services), all participants provided written informed consent prior to the focus group discussion session, and patient confidentiality and good research practices were followed throughout the course of the study.

#### Inclusion/exclusion criteria for the focus groups

Potential participants were assessed by clinicians at each site to determine if they met inclusion and exclusion criteria. Participants were required to: be 18 years of age or older; meet the American College of Rheumatology (ACR) criteria [[1]] for FM as documented in patient charts; report disturbed sleep at least three times per week for at least one month in the three months prior to screening; and to read, speak and understand English. Participants were allowed but not required to be receiving current treatment for FM.

Patients were excluded if: they had a history of a sleep or circadian rhythm sleep disorder as a result of a medical condition other than FM (such as restless leg syndrome, narcolepsy, sleep apnea, or phase advance or delay syndromes) within the past five years; had been on night or rotating shift work, had traveled across more than four time zones in 14 days prior to the focus group, or experienced regular disruptions during the night from another cause (e.g., care for dependents); had severe pain due to other conditions or with any widespread inflammatory musculoskeletal disorder, rheumatic disease (other than FM), active infections, untreated endocrine disorders, or somatoform disorders; or had uncontrolled, severe or unstable depression that could have interfered with the conduct of the study.

#### Procedures

Groups were moderated by an interviewer trained in focus group techniques and participants were remunerated for their time. A semi-structured discussion guide was developed specifically for this study based on the literature review and the discussions with experts (see Additional file 2 online). The sessions were audio-recorded with participant permission and later transcribed for analysis. The topics, questions, probes, and group activities were designed to focus broadly on patients’ perspectives of their FM as it related to their experiences with sleep, their overall experiences with sleep, the frequency and variability of symptoms, and the perceived importance of their symptoms or experiences. Focus groups lasted approximately 1½ to 2 hours.

After the completion of each focus group discussion, participants completed a brief set of questionnaires to permit description of the characteristics of the participant sample. These questionnaires included the (FIQ) evaluating FM symptoms and impact over the past week, numerical rating scales (NRS) evaluating pain, sleep disturbance and tiredness due to FM over the past week, and the Subjective Sleep Questionnaire (SSQ) which asks more detailed questions about the previous night’s sleep (time to fall asleep, number of awakenings, time awake during the night and length and quality of sleep). All participants had completed a socio-demographic form immediately before the focus group began. In addition, sites completed a brief clinical form for each participant describing their history of fibromyalgia (length of time with diagnosis, key symptoms, and current medications).

#### Analytic approach

Qualitative analysis techniques similar to those outlined by the International Society for Pharmacoeconomics and Outcomes Research (ISPOR) PRO Good Research Practices Task Force Report Part 1 were followed [[6]]. Qualitative data (i.e., transcriptions of discussions) were organized with a qualitative analysis software program, ATLAS.ti (version 5.0), in order to conduct a systematic analysis of the transcripts. A set of thematic codes were drafted based on the interview guide, which was informed by the literature review and expert interviews. This list was used to code the transcript data, and to record new themes arising from the focus group analysis, thus capturing the major topic areas of discussion in the focus groups. After the coding and cleaning (removal of all personal identifiers) of transcripts was completed, they were analyzed by reviewing the specific quotes by code in order to synthesize and summarize the results, as well as to assess saturation of concepts. The primary focus of this qualitative analysis was to identify themes around the experience of sleep disturbance in FM populations, and the qualitative results were summarized by post-hoc assessment of endorsement of concepts per focus group (Table 1) and reporting of the number (and percentage) of participants that endorsed each concept. Saturation is necessary prior to instrument development and is reached when similar themes arise across focus groups and no substantially new information is uncovered in later focus groups [[25]].

**Table 1:** **Saturation grid of sleep disturbance discussion in focus groups**^**1**^

**Concept of Sleep Disturbance**	**FG 1 (n = 9)**	**FG 2 (n = 8)**	**FG 3 (n = 8)**	**FG 4 (n = 5)**	**FG 5 (n = 4)**
**Trouble falling asleep**	X	X	X	X	X
**Wake after sleep onset**	X	X	X	X	X
**Tossing and turning**	X	X	X	X	X
**Waking up due to discomfort**	X	X	X	X	X
**Restfulness of sleep**	X	X	X	X	X
**Trouble staying asleep**	X		X	X	X
**Unrested**	X	X		X	X
**Never fully asleep**	X			X	X
**Time to fall asleep**		X	X		X
**Length of sleep**	X		X	X	
**Cyclical sleep**		X	X	X	
**Level of Energy**	X	X			X
**Unrefreshed**		X			X

^1^An “X” indicates the concept was endorsed by one or more patients within the focus group.

#### Results

Although 36 participants were consented and interviewed, two were found to be ineligible after study completion due to self-reported co-morbid disorders (multiple sclerosis and sleep apnea). All data for these two patients were excluded from analysis; therefore, the sample size for this study is 34.

Participants were primarily female (n = 30, 88.2%) and white (n = 25, 73.5%). The mean age was 47.8 (±11.9) years. Seven were unemployed and nine reported being disabled; of these 16, more than half (n = 10, 62.5%) felt this was due to FM. See Table 2 for demographic characteristics and Table 3 for self-reported sleep disturbances experienced within the previous week as reported on the post-focus group questionnaire.

**Table 2:** Focus group and cognitive interview sample description – demographic characteristics

**Demographic characteristics - mean (SD); Range**	**Focus group (n = 34)**	**Cognitive interview (n = 15)**
**Age** (years)	47.8 (11.9); 22-70	51.4 (10.1); 27-64
**Gender** (n, % female)	30 (88.2%)	14 (93.3%)
**Ethnicity** (n, % Hispanic or Latino)	8 (23.5%)	0 (0%)
Race* (n, %)		
White	25 (73.5%)	11 (73.3%)
Black or African American	1 (2.9%)	3 (20.0%)
Asian	2 (5.8%)	1 (6.7%)
Native Hawaiian or other Pacific Islander	4 (11.7%)	0 (0%)
American Indian or Alaska Native	1 (2.9%)	0 (0%)
Other^1^	6 (17.6%)	0 (0%)
Employment Status* (n, %)		
Employed, full-time	7 (20.5%)	4 (26.7%)
Employed, part-time	9 (26.4%)	1 (6.7%)
Homemaker	2 (5.8%)	1 (6.7)
Student^2^	1 (2.9%)	0 (0%)
Unemployed	7 (20.5%)	3 (20.0%)
Retired	3 (8.8%)	2 (13.3%)
Disabled	9 (26.4%)	6 (40.0%)
Other^2^	2 (2.8%)	0 (0%)
**Unemployed or Disabled due to Fibromyalgia (n, %)**		
Yes^3^	10 (62.5)	7 (77.8%)
No^4^	6 (37.5%)	2 (22.2%)

*Participants could select more than one response option.

^1^Six participants selected other and described themselves as: “Mexican American,” “Russian Jew Spanish,” “Mexican,” “Hispanic/Portuguese,” “Hispanic,” and “Mexican.”

^2^“Pastor”.

^3^One participant checked, “homemaker” and marked, “Yes” for, “If unemployed or disabled, is this due to your fibromyalgia.”

^4^One participant checked, “employed part time” and marked, “no” for “If unemployed or disabled, is this due to your fibromyalgia.”

**Table 3:** Focus group and cognitive interview sample description – self-reported sleep disturbances in the past week

**Sleep characteristic - mean (SD); range**	**Focus group (n = 34)**	**Cognitive interview (n = 15)**
**Sleep disturbance in the past week**	6.4 (1.9);	7.4 (1.4);
(“0 “no sleep disturbance” to 10 “worst possible”)	(1, 10)	(6, 10)
**Pain in the past week**	6.9 (1.8);	7.3 (1.7);
(“0” no pain to “10” worst possible pain)	(2, 10)	(3, 10)
**Tiredness in the past week**	7.4 (1.8);	7.6 (1.7);
(0”not tired to “10” extremely tired)	(2, 10)	(4, 10)
**Time taken to fall asleep last night (in minutes)**	74.5 (69.4);	118.7 (147.4);
	(5, 240)	(5, 600)
**Time spent sleeping last night (in minutes)**	324.0 (76.8);	224 (122.7);
	(180, 510)	(0, 510)
**Number of times awoken last night, (mean, sd)**	2.5 (1.5);	3.3 (2.1);
	(0, 5)	(0, 6)
**Time spent awake after falling asleep last night (in minutes)**	71.4 (166.2);	154 (127.9);
	(0, 960)	(0, 390)
**Quality of sleep**	5.1 (2.3);	2.7 (2.3);
(“0” very poor to “10” excellent)	(0, 10)	(0, 6)

Ninety-seven percent (n = 33, 97%) reported disturbed sleep as a result of FM, mostly attributed to the pain and stiffness, and often drawing negative comparisons between their current sleep and their sleep prior to having symptoms and being diagnosed with FM. One participant noted:

“I said I used to sleep really well. I could sleep all the way through, like before I was diagnosed with fibromyalgia or, you know, even having all the symptoms. I could sleep through the storm. Now anything will wake me up.” (FG 2)

Generally speaking, participants identified a good night’s sleep as being uninterrupted or with minimal interruptions due to physical discomfort. Disturbances relating to both sleep quality and sleep quantity were raised as issues, and it was notable that participants did not consider sleep quality to be directly related to the perceived quantity of sleep*.* Eleven (32%) described insufficient, or simply not enough, sleep as being part of their sleep experience with FM.

“Maybe five, yeah, four or five hours, and there’s many times I wake up at 3:00, 4:00. And that’s not enough…” (FG 1)

Sleep quality was discussed as being the “type” of sleep experienced, with almost one-third (n = 10, 29%) of participants describing not having a good night’s sleep, not experiencing deep sleep, and reporting feelings of restlessness during sleep. They reported awakening during the night due to light sleep or for various other reasons relating to their FM (e.g., pain, sleep in a warped position, discomfort).

“And you can’t get comfortable, you know…. you can never get comfortable, your neck is never in the right position…” (FG 5)

“I just know I wake up, and I-and I’m like, oh, toss and turn. And then I only sleep on my shoulder for maybe like 30 minutes.” (FG 2)

Participants discussed difficulty falling asleep and staying asleep, *“Falling asleep, that takes forever”.* Descriptions included the time it took to fall asleep, being “up every hour,” “frequently waking up,” having difficulty getting back to sleep once awake, being up all night, and awakening earlier than desired.

“I can, initially, go to sleep, but it’s staying asleep that’s very hard. And then, I got to sleep tired and I wake up exhausted, and it’s frustrating.” (FG 4)

“I've become really restless throughout the night. I have trouble falling asleep. And then, I'm kind of in that dozed, as you described, state. You’re never fully asleep. You’re never fully awake. And I can toss and turn and just shift and move.” (FG 4)

Almost half (n = 15) described problems when awakening for the final time in the morning. The most common problems faced when waking up were a feeling of exhaustion, pain and stiffness, and an inability to get out of bed in the morning or get their days started without delay.

“And then you’re staying in bed. I pretty much stay in bed all day.” (FG 5)

“Even if I do get a good night’s sleep, let’s assume like six hours, a good night’s sleep, and I wake up at 6 in the morning, I can’t function to do anything until 12:00 noon. I can’t do the dishes, I can’t do anything, it’s like you’re just-you’re lethargic, you know.” (FG 5)

As part of the review and analysis of the focus group data, participant wording around sleep disturbance was grouped into broader categories. A saturation grid was devised to examine the following concepts in sleep: trouble falling asleep, wake after sleep onset, tossing and turning, waking up due to discomfort, trouble staying asleep, unrested, never fully asleep, time to fall asleep, length of sleep, cyclical sleep (patterns of falling asleep and waking), and feeling unrefreshed (see Table 1).

**Figure 2:**
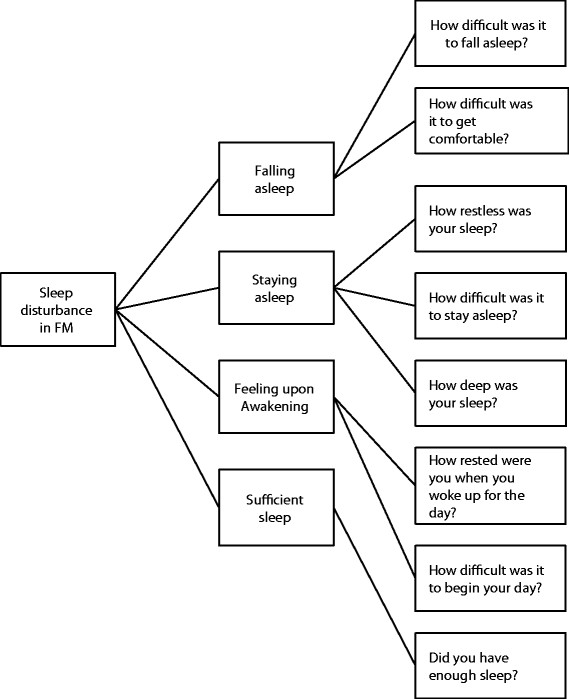
Conceptual framework for sleep disturbance in fibromyalgia.

An iterative process was used to develop a conceptual framework of sleep disturbance in FM, presenting the concepts around sleep disturbance that were reported by the patients as being an important part of the FM experience (see Figure 2).

### Stage 4: Conceptual framework and instrument development

The conceptual framework, based on the results of the literature review, expert interviews, and patient focus groups, informed the development of a daily sleep diary, the Fibromyalgia Sleep Diary (FMSD). An iterative process was used to develop the FMSD, including consideration of results from all stages of this research project and input from all authors. The endorsement of concepts from the focus group was considered (Table 1), in addition to the information about the concepts found within the literature review stage and the expert interview stage (Table 4). The terminology used by the participants during the focus groups was utilized when drafting the content of the FMSD. The FMSD comprises eight items that reflect the sleep disturbances highlighted in the conceptual framework, with wording of the items based on terminology used by participants during the focus groups. Items assess difficulty with falling asleep, restlessness of sleep, difficulty getting comfortable, difficulty staying asleep, degree of deep sleep, degree of being rested when waking up for the day, difficulty with beginning the day, and degree of having enough sleep during the previous night. Responses are on a 0–10 scale with anchors at each end. The FMSD was designed to be completed daily in the morning after final awakening, to aid accurate recall of sleep disturbances, via the telephone using an Interactive Voice Recognition (IVR) System.

**Table 4:** FMSD support

**Concept**	**Hypothesized domain**	**Support from literature review**^**1**^	**Support from Expert interviews**^**2**^	**Support from focus groups**^**3**^	**FMSD Item**	**Support from cognitive interviews (n = 15)**^**4**^
**Difficulty falling asleep, initiating**	Falling asleep	✓	✓	✓	How difficult was it to fall asleep last night?	15/15
**Restless sleep**	Staying asleep	✓	✓	✓	How restless was your sleep last night?	15/15
**Discomfort; difficulty getting comfortable**	Falling asleep	✓	✓	✓	How difficult was it to get comfortable last night?	15/15
**Difficulty staying asleep, maintaining**	Staying asleep	✓	✓	✓	How difficult was it to stay asleep last night?	14/14
**Soundness of sleep**	Staying asleep	✓	✓	✓	How deep was your sleep last night?	15/15
**Waking up, unrested sleep**	Feeling upon awakening	✓	✓	✓	How rested were you when you woke up for the day?	15/15
**Difficulty awakening and arising**	Feeling upon awakening	✓	✓	✓	How difficult was it to begin your day?	15/15
**Quantity of sleep**	Sufficient sleep	✓	✓	✓	Did you have enough sleep last night?	15/15

^1^Identified as important in existing research.

^2^Concept was endorsed by experts during the therapeutic area expert interviews.

^3^Concept was endorsed by patients in the focus group discussions.

^4^Patients understood the item meaning, understood response scale, and were able to select a response.

A daily diary format was chosen for multiple reasons. During the focus groups, participants were able to describe the difference between a night with mild, moderate, or severe sleep disturbance, as well as bad nights versus good nights, indicating day-to-day variability. This diary format with daily recall also matches more objective sleep measures that are often used in clinical trials of insomnia and other sleep disturbances. It avoids the difficulties associated with weekly recall such as 1 or 2 bad nights differentially influencing the weekly recall.

### Stage 5 - Cognitive interviews

#### Rationale

Cognitive interviewing is used to assess the comprehensiveness, relevance, understanding, interpretation and readability of a PRO from the perspective of those intended to complete the measure. The next step of this development process was to conduct cognitive interviews to test the FMSD in FM patients.

#### Methods

Fifteen one-on-one cognitive interviews with FM patients who experienced sleep disturbance were conducted at two community clinical sites located in New Jersey and California. The study was reviewed and approved by Ethical and Independent Review Services IRB, all participants provided written informed consent prior to the interviews, and patient confidentiality and good research practices were followed throughout the course of the study.

#### Inclusion/exclusion criteria

The inclusion and exclusion criteria for the cognitive interviews were the same as those criteria for the focus group study as outlined above. Clinicians at each site completed chart reviews and recruitment interviews to ensure adherence to the inclusion/exclusion criteria. Sites completed a similar clinical form as well.

#### Procedures

Prior to the interview, participants completed the FMSD. To simulate the IVR system administration, during the interview participants completed the FMSD by calling a study team member who read the items and response options using a script and allowed the participant to select responses by pressing the telephone keypad.

Participants then engaged in a standardized retrospective cognitive interview involving detailed questions about each item on the diary. This focused on comprehension and understanding of the FMSD items, including rationale for answer selection, potential variability of answer, understanding of the recall period, questions around redundant items and potential wording changes, and review of alternative response options. Alternative response options included using the “worst possible symptom and best possible symptom” as anchor points as well as “no difficulty and worst possible difficulty”. In addition, the interview assessed ease of completion, comprehensiveness, and relevance of the diary overall and the IVR format.

As in the focus groups, following the interview, participants completed: the FIQ, the weekly pain NRS, tiredness NRS, and the SSQ.

#### Analytic approach

A content analysis approach was used to evaluate the information gathered during the cognitive interviews. The analysis was based on recall, notes taken by the interviewer, audio recordings, and transcripts. The transcribed cognitive interviews were organized with the same qualitative analysis software program, ATLAS.ti (version 5.0), used for Stage 4, in order to conduct a systematic analysis of the transcripts. The qualitative analysis was organized by areas of discussion. A coding dictionary was developed to organize the data and be able to better cluster discussion of the FMSD items. The codes were utilized to identify specific areas of discussion about the diary across participants. For example, an instructions code captured all discussion around participants’ understanding of the FMSD instructions. Output of the coded data was created to permit examination of the transcribed interview data to evaluate participant responses regarding their comprehension, relevance of the FMSD to their experiences, and ease or difficulty with selecting a response for each item on the FMSD. Participant preference for the various alternate options that were presented was also reviewed. The results were used to assess the content validity of the FMSD.

#### Results

The majority of the 15 participants were female (93.3%) with a mean age of 51.4 (10.1) years with a range of 27 to 64 years; 73.3% identified themselves as white, 20.0% as black or African American, and 6.7% Asian. Most participants assessed their overall health as either “fair” (40.0%) or “poor” (40.0%), with two selecting “good” (13.3%) and one “very good” (6.7%). See Tables 2 and 3 for self-reported sleep disturbances.

The FMSD instructions were well understood. Seven of the eight FMSD items were interpreted as intended by all participants. One participant misunderstood the item “How difficult was it to stay asleep?” confusing it with difficulty falling asleep. Participants reported no difficulty with the response options, despite some shifts in direction of the response scale between items (e.g., the response indicating the least amount of impact changes between 0 and 10 depending on the item). In general, the original anchors of “not at all” and “extremely” on a 0 – 10 point scale were preferred to the alternatives that were presented. All participants understood the time frame within each question and reported this as being appropriate to the item.

During discussion of the FMSD item that asks about being rested when waking for the day, participants were asked to consider an alternate term “refreshed.” Responses varied as to whether the two terms were the same or different in meaning. Participant comments did reveal a theme around the term “refreshed” being viewed as an overly positive term and seen as beyond normal rest; therefore, the original wording (i.e., rested) was retained.

“‘Rested’ means that it, the nervous system has calmed down, that you’re um, calm, that there’s nothing there and you feel okay, it’s, whatever it was that you brought to bed with you is gone away. You’re rested. Um, ‘refreshed’ is more like um, it’s beyond rested. You are rested and you are rejuvenated. There’s a ‘plus’ factor in there, just a little more, to me.”

Two alternate phrasing options for the FMSD were presented: starting items with the phrase “Last night,” and presenting the items as statements rather than questions. Presenting items as questions (as opposed to the alternative of statements) was preferred. The majority of participants did prefer adding the phrase “last night” to applicable FMSD items; therefore, “last night” was added to the applicable items (i.e., 1–5 and 8). This was the only change made to the FMSD following the cognitive interviews.

Overall, the FMSD was found to be clear, comprehensible and relevant to patients. Participants were able to interpret the items as intended, understood the terminology used, and agreed that it captured the sleep experiences associated with FM. In addition, participants found that the response scales and recall period were appropriate, and that it was relatively easy to complete through the simulated IVR system.

Table 4 presents a summary of the support for each item in the FMSD from the various stages of the research.

## Conclusions and discussion

Sleep disturbance has clearly been identified as an important part of the experience of fibromyalgia among patients, as seen in the literature [[2]–[4]], Moreover, the qualitative work with both patients and experts in this study confirmed the findings from the literature that the main sleep disturbances experienced within FM relate to problems falling asleep, staying asleep (disturbed sleep and frequent awakenings), how a person feels upon awakening, and the amount of sleep obtained.

The many PRO measures that have been used to assess sleep in FM were compared against the recommendations from the FDA PRO guidance [[5]] and ISPOR PRO task force [[6]]. In general, these PRO measures were nonspecific to FM, and or sleep, and lacked the necessary qualitative evidence to demonstrate relevance to the unique features of sleep disturbance in the FM population. This included a lack of patient input into item development, no demonstration of FM patients’ comprehension of the instruments, and lack of validation in the FM population. Importantly almost all of them were not developed specifically for FM patients

To fill this need, the FMSD was developed, in line with the recommendations in the FDA PRO guidance [[5]] and of the ISPOR PRO Task Force [[6]]. The concepts evaluated within the FMSD were identified from in-depth qualitative work with FM patients, and items were developed using terminology that was derived from the language patients used during the focus groups. Further qualitative work with patients was conducted through cognitive interviews to assess the content validity of the measure. Results showed that the FMSD items were relevant to the FM population with content that was interpreted as intended, and questions and response options that patients understood and could answer.

A limitation of this research is that the FMSD focuses specifically on PRO measurement of sleep disturbance in FM populations; therefore, it is recommended that the FMSD be used as part of a wider measurement strategy for patients with FM, particularly in the context of clinical trials. Sleep disturbance in FM has been shown to have an impact on the functioning and health-related quality of life for FM patients [[3]], which was confirmed in the focus groups conducted in the early stages of this work. These broader areas of daily functioning and quality of life are an important part of evaluating FM as a whole, as they are impacted by all symptoms in FM and not simply by sleep disturbance. Further limitations include the use of a convenience sample focused on clinical trial like eligibility criteria.

The qualitative evidence generated thus far strongly supports the content validity of the FMSD as a PRO measure of sleep disturbance in FM populations. However, additional study of the FMSD is necessary to explore further the psychometric properties of this tool, as outlined within the recommendations for PRO development [[5]].

## Abbreviations

ACR: American college of rheumatology

FDA: Food and drug administration

FG: Focus group

FIQ: Fibromyalgia impact questionnaire

FM: Fibromyalgia

FMSD: Fibromyalgia sleep diary

ISPOR: International society for pharmacoeconomics and outcomes research

IVR: Interactive voice recognition

NIH: National institutes of health

NRS: Numerical rating scales

OMERACT: Outcome measures in rheumatology

PRO: Patient reported outcome

PSQI: Pittsburgh sleep quality index

SD: Standard deviation

SSQ: Subjective sleep questionnaire

## Competing interests

CB, VP, MR, and TS are employed by Pfizer. LK, SM, KH, and KM were all employees of Evidera (formerly a division of United BioSource Corporation) at the time of the study. Evidera received support to complete this manuscript.

TR served as a consultant for Abbott, Accadia, AstraZenca, Aventis, AVER, Bayer, BMS, Cypress, Ferrer, Glaxo Smith Kline, Impax, Intec, Jazz, Johnson and Johnson, Merck, Neurocrine, Novartis, Proctor and Gamble, Pfizer, Purdue, Shire, Somaxon, Transcept. TR also received research support from Cephalon Merck, Transcept. TR served on the Speakers Bureau for Purdue.

LA received grant/research support from Eli Lilly and Company, Pfizer, Forest, Theravance, Takeda, AstraZeneca, Tonix, Sanofi-Synthelabo, Boehringer Ingelheim, Allergan, Novartis. LA served as a consultant/advisory board for Pfizer, Daiichi Sankyo, Theravance, Purdue, Eli Lilly and Company, Sanofi-Synthelabo, Forest, Sepracor, Allergan, Vivus, Boehringer Ingelheim, Organon, Johnson and Johnson, AstraZeneca, Takeda, Grunenthal, Dainippon Sumitomo Pharma, Shire, Toray. LA served on the Speakers Bureau for Eli Lilly and Company, Forest, Pfizer.

## Authors’ contributions

LK served as the Principal Investigator, overseeing the direction of the project, reviewing all results, and developing the sleep diary. SM and KH participated in the implementation of the project by designing the study protocols, collecting data, analyzing the results, and developing the diary. LA and TR participated in the study by providing clinical expertise for protocol design, diary development, as well as helping to draft the manuscript. LK, SM, KH, KM, CB, VP, MR, TS, LA, and TR all participated in the development of the diary. CB, VP, MR, and TS conceived of the study, and participated in its design and coordination and helped to draft the manuscript. All authors read and approved the final manuscript.

## Additional files

## Supplementary Material

Additional file 1: Literature search strategy: embase and MEDLINE (search date 06JAN2011). Click here for file

Additional file 2: Sample probes from the semi-structured focus group discussion guide. Click here for file
