# Supplementary material for: Assessment of sleep in patients with fibromyalgia: qualitative development of the fibromyalgia sleep diary
Source: Health Qual Life Outcomes. 2014 Jul 14;12:111. doi: 10.1186/s12955-014-0111-6 (PMC4110695; doi:10.1186/s12955-014-0111-6)
Supplement: Additional file 1: — Literature search strategy: embase and MEDLINE (search date 06JAN2011). [file s12955-014-0111-6-S1.docx]

**ADDITIONAL FILE 1**

Literature search strategy: Embase and MEDLINE (search date 06JAN2011)

| **No.** | **Query** | **Results** |
| --- | --- | --- |
| **#1** | **'fibromyalgia'**/exp/mj OR **fibromyalgia**:ti | **5995** |
| **#2** | **'sleep disorder'**/exp OR **'sleep'**/exp OR **sleep***:ab,ti | **191644** |
| **#3** | **'interview'**/exp OR **interview***:ab,ti OR **'questionnaire'**/exp OR **questionnaire***:ab,ti OR **survey***:ab,ti OR **'instrument'**/exp OR **instrument***:ab,ti OR **'rating scale'**/exp OR **scale***:ab,ti OR **measure***:ab,ti OR **'measurement'**/exp OR **measurement***:ab,ti OR **assessment***:ab,ti OR **index***:ab,ti OR **diar***:ab,ti OR **inventor***:ab,ti OR **score***:ab,ti OR **'health related quality of life'**/exp OR **'health related quality of life'**:ab,ti OR **'hrql'**/exp OR **'hrql'**:ab,ti OR **hrqol**:ab,ti OR **'hr-qol'**:ab,ti OR **'quality of life'**/exp OR **'quality of life'**:ab,ti OR **qol**:ab,ti OR **'life quality'**/exp OR **'life quality'**:ab,ti OR **'functional status'**/exp OR **'functional status'**:ab,ti OR **'health status'**/exp OR **'health status'**:ab,ti OR **'health outcome'**:ab,ti OR **'health outcomes'**:ab,ti OR **'outcomes research'**/exp OR **'outcome assessment'**:ab,ti OR **'outcome assessments'**:ab,ti OR **'outcomes assessment'**:ab,ti OR **'outcomes assessments'**:ab,ti OR **'outcome measure'**:ab,ti OR **'outcome measures'**:ab,ti OR **'outcomes measure'**:ab,ti OR **'outcomes measures'**:ab,ti OR **'outcome study'**:ab,ti OR **'outcome studies'**:ab,ti OR **'outcomes study'**:ab,ti OR **'outcomes studies'**:ab,ti OR **'outcome research'**:ab,ti OR **'outcomes research'**:ab,ti OR **'outcome assessment'**/exp OR **'treatment outcome'**/exp OR **'treatment outcome'**:ab,ti OR **'treatment outcomes'**:ab,ti OR **'treatment effectiveness'**:ab,ti OR **'treatment efficacy'**:ab,ti OR **'rehabilitation outcome'**:ab,ti OR **'outcome measurement'**:ab,ti OR **'outcome measurements'**:ab,ti OR **'outcomes measurement'**:ab,ti OR **'outcomes measurements'**:ab,ti OR **'functional assessment'**/exp OR **'functional assessment'**:ab,ti OR **'evaluation'**/exp OR **'evaluation studies'**:ab,ti OR **'patient satisfaction'**/exp OR **'patient satisfaction'**:ab,ti OR **'patient reported'**:ab,ti OR **'self report'**/exp OR **'self report'**:ab,ti OR **'self reported'**:ab,ti OR **'self reporting'**:ab,ti OR **'patient preference'**:ab,ti OR **'patient preferences'**:ab,ti OR **'patient assessment'**:ab,ti OR **'patient assessments'**:ab,ti OR **'patient assessed'**:ab,ti OR **'self evaluation'**:ab,ti OR **'self evaluations'**:ab,ti OR **'patient rating'**:ab,ti OR **'patient ratings'**:ab,ti OR **'patient rated'**:ab,ti OR **'self-completed'**:ab,ti OR **'self-administered'**:ab,ti OR **'self assessment'**:ab,ti OR **'self assessments'**:ab,ti OR **'patient based rating'**:ab,ti OR **'patient based outcome'**:ab,ti OR **'self evaluation'**/exp OR **'well-being'**:ab,ti | **5176353** |
| **#4** | **'animal'**/exp NOT **'human'**/exp | **1223025** |
| **#5** | **#1** AND **#2** AND **#3** NOT **#4** NOT (**letter**:it OR **note**:it OR **editorial**:it) AND [english]/lim AND [2003-2011]/py | **442** |

Literature search strategy: Clinicaltrials.gov (search date 31JAN2011)

| **No.** | **Query** | **Results** |
| --- | --- | --- |
| **#1** | **conditions = fibromyalgia** AND  **outcome measures = sleep** AND  **age group = Adult, Senior** | **49** |

Note: The NIH clinicaltrials.gov website includes both US and internationals trials. The website lists a “ClinicalTrials.gov Identifier number,” as well as “Other Study ID Numbers” which does included ID numbers that link to the International Standard Randomised Controlled Trial Number.
