# Supplementary material for: Assessment of sleep in patients with fibromyalgia: qualitative development of the fibromyalgia sleep diary
Source: Health Qual Life Outcomes. 2014 Jul 14;12:111. doi: 10.1186/s12955-014-0111-6 (PMC4110695; doi:10.1186/s12955-014-0111-6)
Supplement: Additional file 2: — Sample probes from the semi-structured focus group discussion guide. [file s12955-014-0111-6-S2.docx]

**ADDITIONAL FILE 2**

Sample Probes from the Semi-Structured Focus Group Discussion Guide

| 1. **INTRODUCTION**     1. Please introduce yourselves    2. Tell us when you were diagnosed with fibromyalgia and what led you to seeking a doctor to get a diagnosis.    3. How would you describe your sleep? |
| --- |
| 1. **GENERAL SLEEP DISTURBANCE DISCUSSION**   *(note: in order to be eligible subjects must have recently experienced sleep disturbance)*   - 1. What kind of difficulty with sleep do you experience?   2. How do you think your sleep disturbance relates to your FM?   3. Are you able to distinguish between sleep disturbances caused by your FM versus other things?   4. Are there any specific FM treatments that have impacted your sleep? If so, how was your sleep impacted (e.g., better, worse, how)?   5. Do you talk with your nurse or doctor about your difficulty with sleep? If so, what aspects? How do you describe your experience to the doctor/nurse?   6. How do you describe your experiences with FM related sleep difficulty to your family/friends? Or someone who was not familiar with it?   7. Tell us how your life has changed since you have started experiencing FM related sleep difficulty.   8. How do you describe sleep difficulty in terms of…      1. how often you notice problems?      2. how much it bothers you?      3. the impact it has on your life and others?   9. Does light, noise, smell or movement impact your sleep? How so? |
| 1. **SLEEP DISTURBANCE DESCRIPTION – TYPICAL NIGHT & DAY**    1. Now describe for me a typical ***night*** when you experience sleep problems.       1. What do you do to get sleep? What do you do to stay asleep? What words or phrases do you use to describe a night when you experience sleep problems to others?    2. How would you describe a “***good***” nights’ sleep?    3. Now for the opposite question, how would you describe a “***bad***” nights’ sleep?    4. Now describe for me a typical ***day*** when you’ve experienced sleep problems? |
| 1. **SLEEP DISTURBANCE IMPACT (FOCUS ON DAYTIME IMPACT)**    1. In your opinion, what *FM symptoms* most *impact your sleep*?    2. Please describe how your sleep difficulty impacts your life *(e.g., affects the things you do during the day/or “your day” in general)*? |
| 1. **FATIGUE AND TIREDNESS**    1. Please describe what the term *fatigue* means to you.    2. Please describe what the term *tiredness* means to you.    3. How are fatigue and tiredness the same or different? |
| 1. **SLEEP DISTURBANCE – VARIANCE:**     1. How often do you experience sleep disturbance?    2. Are there certain times of night/day when your sleep disturbance symptoms are better/worse? *(e.g., first thing in the morning? Mid-day? Late at night?)*    3. How does your sleep disturbance change over time?    4. How does the way sleep difficulty impacts your day change over time? |
| 1. **SLEEP DISTURBANCE –RECALL PERIOD**    1. In discussing sleep, are you able to recall your sleep from last night? Two nights ago, three nights ago, over the last week? Over the past two weeks? |
| 1. **SLEEP DISTURBANCE –RESPONSE OPTIONS**    1. How would you rate your sleep difficulty? *(e.g., mild, moderate, severe, 0-10, etc.)*    2. Describe how you would define *severe* sleep difficulty?    3. Describe how you would define *moderate* sleep difficulty?    4. Describe how you would define *mild* sleep difficulty? |
